# Impact of different oral treatments on the composition of the supragingival plaque microbiome

**DOI:** 10.1080/20002297.2022.2138251

**Published:** 2022-10-31

**Authors:** Alexander Rabe, Manuela Gesell Salazar, Stephan Michalik, Thomas Kocher, Harald Below, Uwe Völker, Alexander Welk

**Affiliations:** a Interfaculty Institute for Genetics and Functional Genomics, Department of Functional Genomics, University Medicine Greifswald, Felix-Hausdorff-Str. 8, 17475 Greifswald, Germany; b Center for Dentistry, Oral and Maxillofacial Medicine, Department of Restorative Dentistry, Periodontology, Endodontology, and Preventive and Pediatric Dentistry, Dental School of University Medicine Greifswald, Fleischmannstraße 42-44, 17489; c Institute for Hygiene and Environmental Medicine, University Medicine Greifswald, Walter-Rathenau-Straße 49 A 17475 Greifswald, Germany

**Keywords:** Teeth, dental plaque, metaproteomics, healthy human oral microbiome, lactoperoxidase, essential oil, nLC-MS/MS

## Abstract

**Background:**

Dental plaque consists of a diverse microbial community embedded in a complex structure of exopolysaccharides. Dental biofilms form a natural barrier against pathogens but lead to oral diseases in a dysbiotic state.

**Objective:**

Using a metaproteome approach combined with a standard plaque-regrowth study, this pilot study examined the impact of different concentrations of lactoperoxidase (LPO) on early plaque formation, and active biological processes.

**Design:**

Sixteen orally healthy subjects received four local treatments as a randomized single-blind study based on a cross-over design. Two lozenges containing components of the LPO-system in different concentrations were compared to a placebo and Listerine®. The newly formed dental plaque was analyzed by mass spectrometry (nLC-MS/MS).

**Results:**

On average 1,916 metaproteins per sample were identified, which could be assigned to 116 genera and 1,316 protein functions. Listerine® reduced the number of metaproteins and their relative abundance, confirming the plaque inhibiting effect. The LPO-lozenges triggered mainly higher metaprotein abundances of early and secondary colonizers as well as bacteria associated with dental health but also periodontitis. Functional information indicated plaque biofilm growth.

**Conclusion:**

In conclusion, the mechanisms on plaque biofilm formation of Listerine® and the LPO-system containing lozenges are different. In contrast to Listerine®, the lozenges led to a higher bacterial diversity.

## INTRODUCTION

Starting from birth, bacteria colonize the human supra-organism and have an enormous influence on the development of the immune system and thus on human health status [1]. Next to the gut, the second most complex bacterial ecosystem is the oral microbiome [2,3]. Besides the planktonically living bacteria in saliva, the bacteria in oral biofilms are of special interest [4,5]. Biofilms are defined as a community structure of microorganisms living in a matrix of synthesized exopolysaccharides [6,7]. The mechanical stability of the matrix and its high bacterial diversity lead to synergistic interactions, e.g. the extension of the genetic repertoire by horizontal gene transfer within the biofilm [4,8]. This organizational structure enables the biofilm to show a special resistance to external environmental influences such as nutrient limitation, the human immune system and antibiotics [6].

In principle, supragingival plaque is assumed to have a positive role, since it serves as a barrier against the colonization of pathogens [9]. However, a diet with a high carbohydrate content [10] combined with poor oral hygiene can lead to a bacterial shift [11,12] and cause diseases such as dental caries [13] or periodontitis [14]. Saliva is part of the 1st line defense against a dysbiotic biofilm, because it mechanically removes bacteria [12] but it also contains components of the innate immune system such as lactoperoxidase (LPO) [15,16].

The salivary glands produce among others the enzyme lactoperoxidase, which catalyzes an ionic substrate such as thiocyanate (SCN^−^) in the presence of H_2_O_2_ to form a highly reactive anti-microbial oxidation product, hypothiocyanite (OSCN^−^) [10,16,17]. Since the 1980s, an increased knowledge of the LPO system has been leading to toothpastes or mouthwashes, which contain components of the LPO system to support the natural antimicrobial defense process [16,18]. The applicability of LPO products poses a challenge, as they cannot be used during the day between meals due to their volumes (mouth rinse) or additional materials like toothbrushes [18]. Furthermore, most of the antimicrobial substances used in oral health care products affect the whole microbiome. This means that all bacteria including the oral commensal flora will be reduced. However, it would be better if the commensal flora would not be reduced or even better, supported. Therefore, human own defense systems, such as the LPO system in saliva, are of interest.

Clinical studies provide insight into the effectiveness of the products, e.g. by performing plaque regrowth studies using traditional microbiological techniques [19]. Such a standard cross-over plaque-regrowth study [20] demonstrated that mouth rinse Listerine® Total Care™ (A – positive control) was statistically significantly more effective than the LPO-system-lozenges (B- 0.083% H_2_0_2_ accordingly a 1:2 H_2_0_2_/SCN- relation), (C- 0.04% H_2_0_2_ accordingly a 1:4 H_2_0_2_/SCN- relation), and the placebo lozenge (D) in inhibiting plaque. Listerine® rinse (A) as well as Lozenges (B) and (C) were statistically significantly more effective than the placebo lozenge (D), but no statistically significant differences could be observed between them.

However, studies based on traditional microbiological evaluation techniques cannot address the effects of these treatments on the composition of the biofilm. Proteomics in combination with habitat-specific taxonomic and genomic databases allows studies of biofilms without the cultivation of bacteria and allows in-depth investigation of the behavior and composition of a biofilm directly in its natural habitat [21–25]. Thus, metaproteomic approaches that monitor changes at the protein level and their impact onto metabolic pathways within the bacterial community should be used in a complementary manner to improve the understanding of the microbiome by monitoring changes of gene expression [26] and to develop more personalized ways to positively support existing natural mechanisms of plaque control [27].

In this pilot study, we used an established metaproteomic approach [28,29] to evaluate the effect of the lozenges used in Welk et al. [19] on the microbiome composition and the changes at the protein level with respect to their functions in metabolic pathways in the bacterial community. To the best of our knowledge, this is the first study combining a well-recognized and established clinical model in dentistry [20] with a metaproteomic study [30].

The results of both studies are expected to support our long-term goal to develop a lozenge, which can be used as an easily applicable addition to daily oral hygiene, to positively influence the microbiome composition to ensure that commensal, non-pathogenic bacteria are the dominant species in the plaque biofilm.

## Material and methods

This complementary study received a positive vote by the ethics committee of the University Medicine Greifswald and was conducted in accordance with the recommendations of the Declaration of Helsinki from 1996. The clinical trial was registered in the German Database for clinical trials (DRKS00022810, date of registry: 02.09.2020).

### Clinical study design

**Figure 1.**
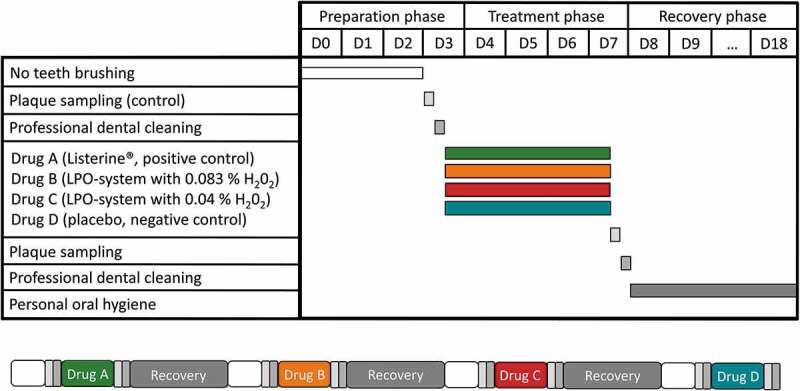
Study design of a 4-day plaque regrowth clinical model. In this randomized single-blinded study two sugar-alcohol based drugs containing high (Drug B – 0.083% H_2_0_2_ accordingly a 1:2 H_2_0_2_/SCN^−^ relation) and low (Drug C – 0.04% H_2_0_2_ accordingly a 1:4 H_2_0_2_/SCN^−^ relation) concentrations of components of the LPO system were evaluated regarding their influence on the plaque microbiome. Drug A, an essential oil containing mouth rinse, served as the positive control and Drug D based only on sugar-alcohols, as the placebo. Each cycle started with a preparation phase (D0-D3) without any kind of oral hygiene, followed by one treatment (D3-D7) and a recovery phase (D7-D18) of at least ten days. The study was also designed as a four-replicate cross-over study, where each subject was his or her own control.

The design of the 4-days standard randomized plaque-regrowth study [19,20] is displayed in Figure 1. All 16 study participants (six male and ten female) were oral healthy dental students of the Greifswald dental school, who gave their written informed consent for this study. The participants were between 20 and 30 years old with a mean age of 23.4 years.

Both test lozenges were based on sugar alcohols (xylitol, sorbitol, mannitol) and contained all components of the LPO system (10 mg LPO 350 U/mg (Sternenzym, Germany), 7.5 mg KSCN) and H_2_0_2_ either in high concentrations (Drug B: 0.083% H_2_0_2_ accordingly a 1:2 H_2_0_2_/SCN^−^ relation) or low concentrations (Drug C: 0.04% H_2_0_2_ accordingly a 1:4 H_2_0_2_/SCN^−^ relation). For Drug B and Drug C, carbamide peroxide (CPO) was used as the H_2_O_2_ donor, because it is very stable and releases H_2_O_2_ in a graduated way [31]. Drug D (placebo) was also a lozenge and had the same composition as Drug B and Drug C without the components of the LPO system. Drug A was Listerine® Total Care™ (Johnson & Johnson GmbH, Neuss, Germany) and is a commercially available mouth rinse containing essential oils. Using Drug A and Drug D as positive and negative control, allowed the results of Drug B and C to be attributed to the effect of two different hygiene measures and the LPO system.

In addition, each cycle started with a preparation phase followed by the treatment and a final recovery phase of at least 10 days. First, in the preparation phase, the participants suspended any kind of personal oral hygiene (timepoint: D0) for 3 days to support the recovery of the oral microbiome. On the fourth day (timepoint: D3) supragingival plaque was collected and served as the control sample. To ensure that plaque was totally removed, a professional dental cleaning was performed by the study dentist followed by the treatment phase (timepoints: D3 – D7), where the volunteers received one of the four drugs.

The mouth rinse solution (Drug A) had to be used twice daily in the morning and evening according to the manufacturer’s instructions. The Drugs B-D were sucked five times daily every 3 hours between every 8 o’clock am and 8 o’clock pm for a period of 10–15 minutes.

On the last day of the treatment phase (D7), plaque that had built up during treatment was collected and the teeth were professionally cleaned. The recovery period of at least 10 days began and the participants resumed their personal oral hygiene (Figure 1).

### Dental plaque collection procedure

**Figure 2.**
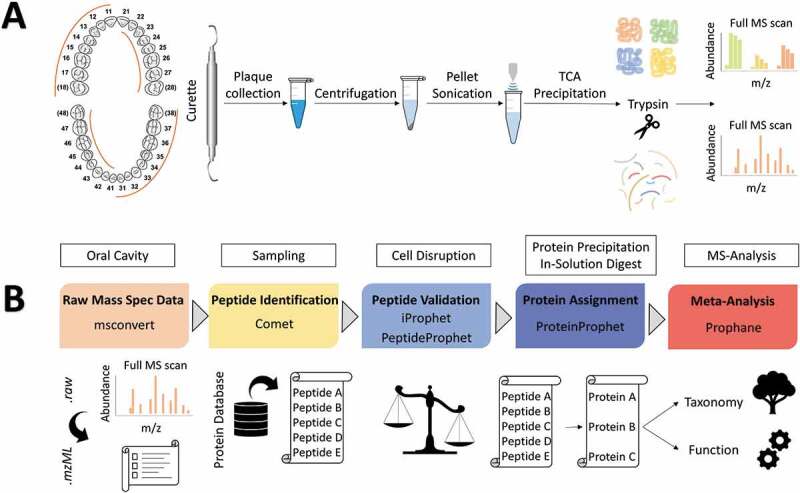
Workflow for supragingival plaque collection and preparation for nLC MS/MS Measurements (A). Collection of dental plaque in sterile 1x PBS from all four quadrants of the human mouth from at least 24 teeth using a sterile curette. After centrifugation, the pellet was resuspended in the TE buffer and treated with ultrasound. The protein mixture was precipitated with TCA and digested using trypsin to measure the peptide mixtures on a Q Exactive™ Plus in DDA mode.

Using a sterile curette (Universal Curette, Hu-Friedy Mfg. Co. LLC, Frankfurt am Main, Germany), supragingival plaque was collected from at least 24 tooth surfaces of all four quadrants (Figure 2A). The curette with the collected plaque was transferred several times during sample collection to a sterile tube (SafeSeal micro tubes, Sarstedt AG & Co., Nümbrecht, Germany) containing 3 ml sterile 1x PBS (Life Technologies GmbH, Darmstadt, Germany) and shaken until the plaque was detached from the curette. Finally, the sample material was vortexed for 30 s to create a suspension. In the next step, 20 µl of a protease inhibitor (Sigma Aldrich, St. Louis, MO, USA; v/v 1:20) per 1 ml sample volume was added and samples were centrifuged for 3 min at 6,200 g and 4 C^°^. The remaining pellets were immediately frozen in liquid nitrogen and finally stored at −80°C.

Spectral processing and metaproteome annotation (B). The open-source software Trans-Proteomic Pipeline processed raw spectra of nano LC-MS/MS measurement. Peptides were identified based on the Comet algorithm and filtered regarding their FDR to increase the validity of peptides. A combined database consisting of human protein sequences from UniProt and bacterial protein sequences of the human oral microbial database provided the basis for protein identification Taxonomic classification, functional prediction and relative quantification was performed by Prophane. Figure 2 is adapted from Rabe et al. [35,36].

### Dental Plaque sample preparation and nLC MS/MS Measurement

The pellets were resuspended with 300 µl TE buffer (10 mM Tris; 1 mM EDTA; pH 8.0). Subsequently, the biofilm and its cells were disrupted by an ultrasound treatment (Labsonic U – B. Braun Melsungen AG, Melsungen Germany) on ice for 3 × 30 s and 50% power of the device to release the proteins. Cell debris and the cytosolic proteins were separated by centrifugation (30 min, 4 C°, 16,200 g). The supernatant containing proteins was transferred to a new tube and stored on ice. The preparation of the protein mixture for the nLC-MS/MS measurement and the method for the mass spectrometric measurement were already described in detail [28,29]. Briefly, proteins were precipitated by TCA and washed several times with acetone. The vacuum dried pellet consisting of precipitated proteins was dissolved in 30 µl 8 M/2 M urea/thiourea solution. The protein concentration was determined with a Bradford Assay (Bio-Rad Laboratories GmbH, Munich, Germany). Cysteines were reduced with dithiothreitol (DTT) and alkylated with iodoacetamide IAA) with subsequent digestion of 4 µg of the protein mixture using trypsin. The resulting peptide mixture was purified after a digestion period of 17 h by ZipTipC18 material (Merck KGaA, Darmstadt, Germany). Finally, 2 µg of peptides were separated with a reverse phase nano HPLC Ultimate® 3000 Nano HPLC (Thermo Scientific) and analyzed on a Q Exactive™ Plus (Thermo Scientific) in data-dependent mode.

### Metaproteome assembly, mapping, and annotation

We designed a workflow based on open-source software applications to analyze our metaproteomic datasets, described in detail in one of our earlier metaproteome studies [28,29]. Figure 2B provides an overview of the most important steps and is briefly described subsequently. To evaluate our 128 MS/MS measurements, we used the Trans-Proteomic Pipeline (http://tools.proteomecenter.org/software.php) [32,33] and have chosen the Comet MS/MS search algorithm (http://comet-ms.sourceforge.net/) [34,35] for peptide and protein identification, based on a combined database with 1,079,744 bacterial sequences of the human oral microbial database (HOMD, www.homd.org) [36,37] and 20,154 human sequences from UniProt (UniProtKB/Swissprot, www.uniprot.org) [38]. Peptides and proteins were filtered according to their iProphet probability at 0.05 Protein FDR (iProphet iProb = 0.9015). Using an R script (version: 4.1.1) [39], only proteins identified with at least one unique peptide were used for further analysis. Finally, proteins were classified taxonomically using the Lowest-Common-Ancestor algorithm (LCA) [40] and regarding their functional TIGRFAM assignment (TIGRFAM library version 15.0; e-value: < 0.01) [41] by the bioinformatic pipeline Prophane (www.prophane.de) [42,43]. All proteins were relatively quantified using normalized spectral abundance factor (NSAF) values [44].

The measured MS/MS data of our study were uploaded to the publicly accessible MassIVE database (dataset name: MSV000089755; doi:10.25345/C57D2QB93).

### Statistical analyses

The statistical calculations as well as the image creations were performed with R (version: 4.1.1) supported by the R Foundation for statistical computing [39].

The NSAF values for each treatment sample have been median normalized to their corresponding control. Values of treatments were divided by control values for each of the ratio calculations, whereby missing or infinite values were not considered.

We selected at minimum 50% valid values per sample for a paired two-sided Wilcoxon signed rank test, which was performed with a set confidence interval of 0.95. To detect significant changes, the cutoff was set to the fold-change = 1.5 and for the p-value = 0.05. Significant results are presented in Volcano and Violin plots created using different R packages (Supplemental Table 4).

The influence of treatments on the taxonomic composition of the plaque microbiome was visualized using the metacoder package (Supplemental Table 4) [45]. The natural logarithm of the ratios between treatments and controls before treatment (color scale) was plotted against the summed spectral counts (thickness of taxonomic clades).

## Results

### Spectral processing results

For our complementing 4-day plaque regrowth study, 4 µg protein of total plaque of 0.88–2.6 (median QHI; Oral hygiene index according to Quigley–Hein) was prepared from each of the 128 samples and analyzed by mass spectrometry using a Q Exactive Plus (Thermo Scientific). The MS/MS analyses of the whole sample set resulted in 5.4 million spectra, of which 2.5 million spectra (identification rate: 46.3%) could be assigned based on our database consisting of human and bacterial protein sequences. Across all samples, a total of 124,101 distinct peptides could be identified with a pepFDR ≤1.56%, thereof 106,980 were of bacterial and 17,121 of human origin.

At the protein level, we only considered proteins that had a protFDR ≤5.0% and contained at least one unique peptide to minimize the possibility of misclassification. Based on these quality criteria, an average of 1,916 (± 465) metaproteins of bacterial origin as well as 442 (± 171) human proteins were covered per sample.

Analyzing the same protein amount (4 µg) of plaque sample for the metaproteomic analysis, on average 23.5% less metaproteins, were observed after treatment (Ø 1057 metaproteins) with Listerine® compared to the negative control before the treatment (Ø 1382 metaproteins) at the genus level (Supplementary Figure 1). Accordingly, these proteins also covered a lower number of bacterial genera. This contrasts with slightly increased metaprotein numbers in treatment groups B (before treatment: Ø 1304 metaproteins; after treatment Ø 1425 metaproteins) and C (before treatment: Ø 1387 metaproteins; after treatment Ø 1486 metaproteins).

Relative quantification of the metaproteome data was performed using spectral counts, which were used to calculate the NSAF values for each protein. For Drug A (Listerine®, positive control), the relative metaprotein abundance of bacterial proteins per sample decreased from an average of 74.1% before the treatment to an average of 59.1% after the treatment, because Listerine® reduced the bacterial biofilm in general. Correspondingly, the relative abundance of human proteins increased. For both LPO component-based (10 mg LPO 350 U/mg (Sternenzym, Germany), 7,5 mg KSCN) lozenges Drug B (0.083% H_2_0_2_; 1:2 H_2_0_2_/SCN^−^ relation) and Drug C (0.04% H_2_0_2_; 1:4 H_2_0_2_/SCN^−^ relation) as well as for D (placebo), the relative abundance of bacterial metaproteins remained constant with values averaging between 69.0% and 78.7% before and after treatment, indicating that the LPO-based lozenges had no decreasing effect on the bacterial biofilm in general.

### Taxonomic profile and changes at genus level

**Figure 3.**
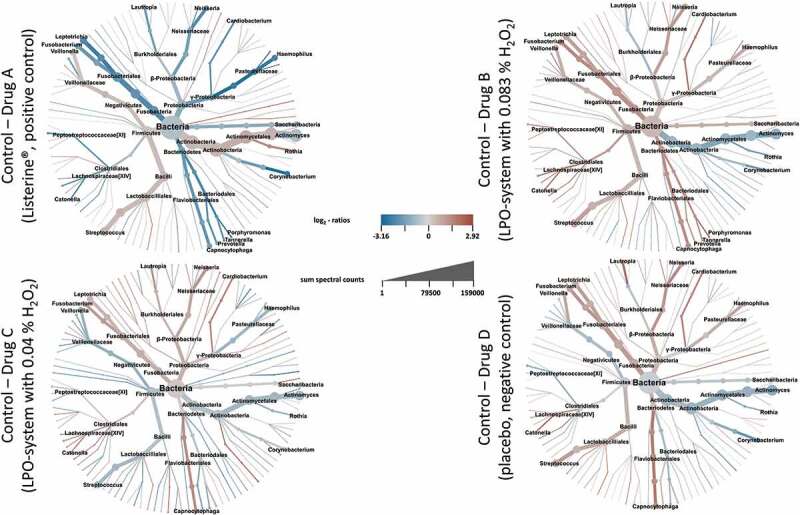
Illustration of the taxonomic diversity as well as the changed bacterial composition for each treatment. The log_2_ median of pairwise NSAF ratios is the basis for the coloring and thus the rate of change. Branch thickness indicates the number of identified spectral counts.

To provide a general overview of the impact of treatments on the diversity of the plaque microbiome, we calculated the ratio of metaprotein abundances between the control and treatment time points by dividing the median normalized NSAF values for each treatment (D7) by its corresponding control (D3) and plotted them against the summed spectral counts in heat map trees (Figure 3).

Across all 128 samples, the metaproteins could be taxonomically assigned to a total of eight phyla, with the phyla *Actinobacteria, Firmicutes, Fusobacteria, Proteobacteria*, and *Bacteriodetes* dominating the composition of the plaque microbiome. *Spirochaetes, Synergistetes*, and *Saccharibacteria* played a minor role. At the genus level, the study covered metaproteins assigned to 116 genera across all samples and the high diversity remained constant after the different treatments in comparison to the control time points.

**Figure 4.**
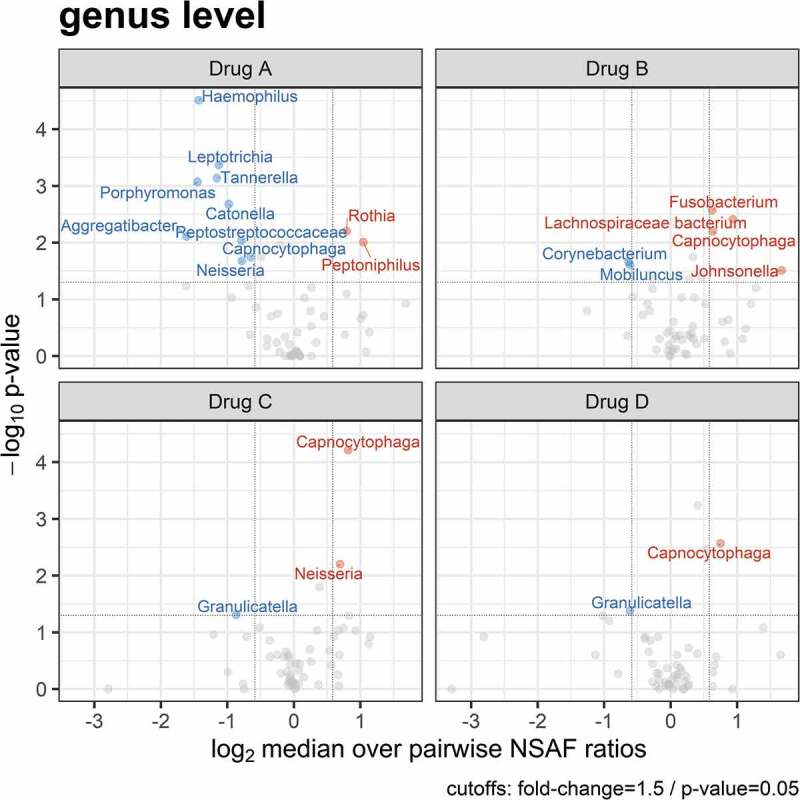
Volcano plots showing significant changes of a paired two-sided Wilcoxon signed rank test (confidence interval = 0.95) for the metaprotein abundances assigned on the genus level for all four treatments. Blue indicates a significant reduction in relative metaprotein abundance after treatment, red indicates a significant increase, and gray indicates no significant changes in relative metaprotein abundance after treatment.

To evaluate whether the treatments caused changes in metaprotein abundances and thus altered plaque microbiome composition, we performed a paired two-sided Wilcoxon signed rank test with a confidence interval of 0.95 (cut-offs: fold-change = 1.5; p-value = 0.05) for genera that occurred in at least 50% of all samples. The Volcano (Figure 4) and violin plot (Supplemental Figure 2) show these significant metaprotein changes at the genus level. Figure 4 shows that Drug A (Listerine®, positive control) primarily led to a significant reduction of the relative abundance for metaproteins of the nine genera, such as *Haemophilus, Leptotrichia* or *Tannerella*, whereas higher metaprotein abundances could be identified for *Rothia* and *Peptoniphilus*. In contrast, the relative metaprotein abundances for the five genera *Fusobacterium, Lachnospiraceae bacterium, Capnocytophaga* and *Johnsonella* increased under Drug B (0.083% H_2_0_2_; 1:2 H_2_0_2_/SCN^−^ relation). However, lower metaprotein abundances could be identified for the genera *Corynebacterium* and *Mobiluncus*.

Treating the subjects with Drug C (0.04% H_2_0_2_; 1:4 H_2_0_2_/SCN^−^ relation) had a similar influence on the plaque metaproteome in comparison to the treatment with Drug D (placebo). Both treatments resulted in a significant decrease of metaprotein abundances for *Granulicatella*, as well as a higher abundance of *Capnocytophaga*. Additionally, an increased metaprotein abundance for the genus *Neisseria* occurred for Drug C.

### Taxonomic changes at species level

**Figure 5.**
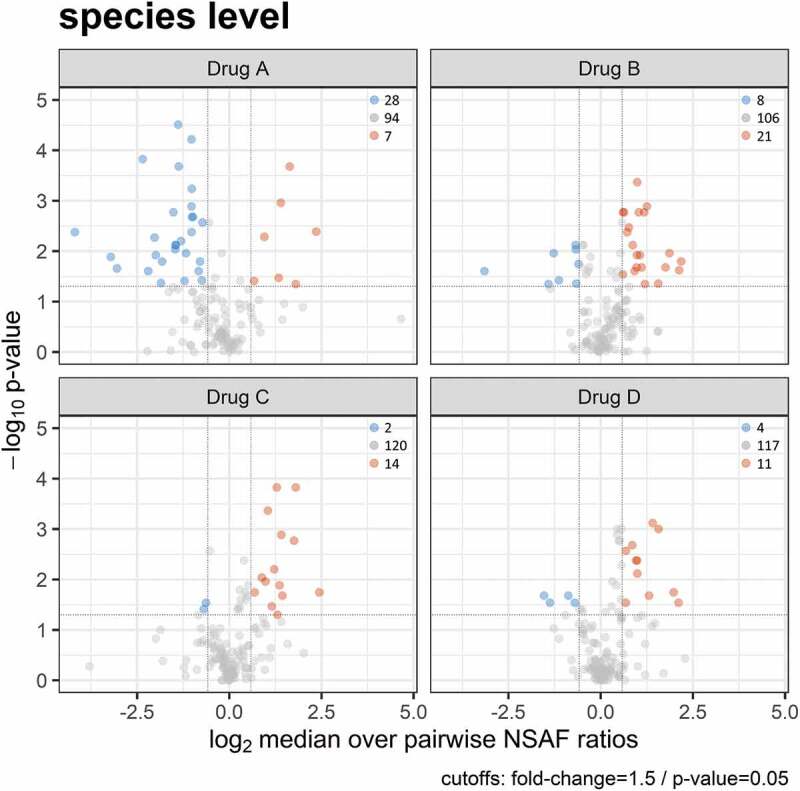
Volcano plots showing significant changes of a paired two-sided Wilcoxon signed rank test (confidence interval = 0.95) for the metaprotein abundances assigned on the species level for all four treatments. Blue indicates a significant reduction in relative metaprotein abundance after treatment, red indicates a significant increase, and gray indicates no significant changes in relative metaprotein abundance after treatment.

To learn more about the effects of the four treatments on the plaque microbiome and its metaproteome, we performed further analyses at the species level, as these are of particular interest from the clinical perspective of dentists. In total, 9,729 metaproteins could be assigned to 351 species and were analyzed for changes in relative abundance. We performed a paired two-sided Wilcoxon signed rank test (confidence interval = 0.95; cut-offs: fold-change = 1.5; p-value = 0.05) for species that occurred in at least 50% of all samples, i.e. the same parameters as for the genus level analyses. Based on the results of the statistical test, metaprotein abundances of 65 species showed significant changes (Figure 5).

Under the treatment of Drug A (Listerine®, positive control) metaprotein abundances for 28 and 7 species were lower and higher, respectively, than in the controls (Supplemental Table 1). Metaproteins of *Rothia dentocariosa* showed the greatest increase in abundance and *Aggregatibacter aphrophilus* the greatest decrease. *Leptotrichia* was the most represented genus with seven species, all of them displaying a reduction in abundance.

For Drug B (0.083% H_2_0_2_; 1:2 H_2_0_2_/SCN^−^ relation), metaprotein abundances for 21 and 8 species were present in higher and lower abundance, respectively, in comparison to the control before treatment (Table 1). The metaprotein abundances with the greatest decrease were identified for *Cronobacter sakazakii* and with the greatest increase for *Lachnospiraceae bacterium ACC2*. All five different *Fusobacteria* showed an increase in metaprotein abundances.

**Table 1.** Summary of significant changed metaprotein abundances and their taxonomic assignment on the species level under Drug B (0.083% H_2_0_2_ accordingly a 1:2 H_2_0_2_/SCN^−^ relation) and Drug C (0.04% H_2_0_2_ accordingly a 1:4 H_2_0_2_/SCN^−^ relation). For each species, their association with healthy and/or diseased oral conditions is given and is color-coded for visual support.

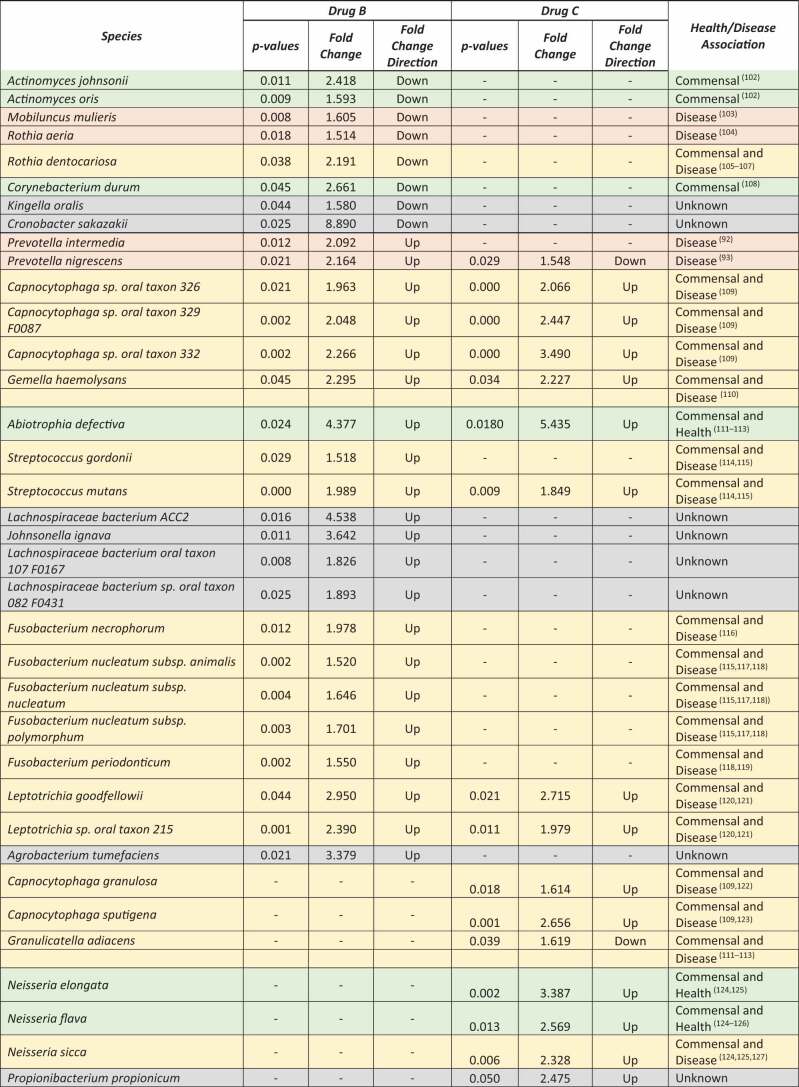

Color legend: green – commensal or health associated; yellow – commensal and disease associated; red: disease associated; grey: no information available if the species is commensal, health or disease associated

For Drug C (0.04% H_2_0_2_; 1:4 H_2_0_2_/SCN^−^ relation) and Drug D (placebo), metaprotein abundance changes were identified for 16 and 15 species, with metaproteins of 14 species showing higher abundance for Drug C (Table 1) and metaproteins of 11 species for Drug D (placebo) (Supplemental Table 2). Both treatments had the greatest similarities among the four treatments by sharing six species with a significant change of metaprotein abundances. Four of the six species originate from the genus *Capnocytophaga* and showed an increased metaprotein abundance, as well as *Neisseria flava* and *Leptotrichia sp. oral taxon 215*.

We observed significant metaprotein changes for *Capnocytophaga sp. oral taxon 329 F0087* and *Leptotrichia sp. oral taxon 215* during all four treatments.

In summary, our findings for the four treatments at the species level were consistent with the analysis results at the genus level. Drug A (Listerine®, positive control) showed a tendency to reduce the metaprotein abundances for most of the species, whereas drug B (0.083% H_2_0_2_; 1:2 H_2_0_2_/SCN^−^ relation) tended to increase it. Drug C (0.04% H_2_0_2_; 1:4 H_2_0_2_/SCN^−^ relation) as well as Drug D (placebo) also showed less pronounced effects on the metaprotein abundances on the species level.

### Bacterial functional profile of the plaque biofilm

**Figure 6.**
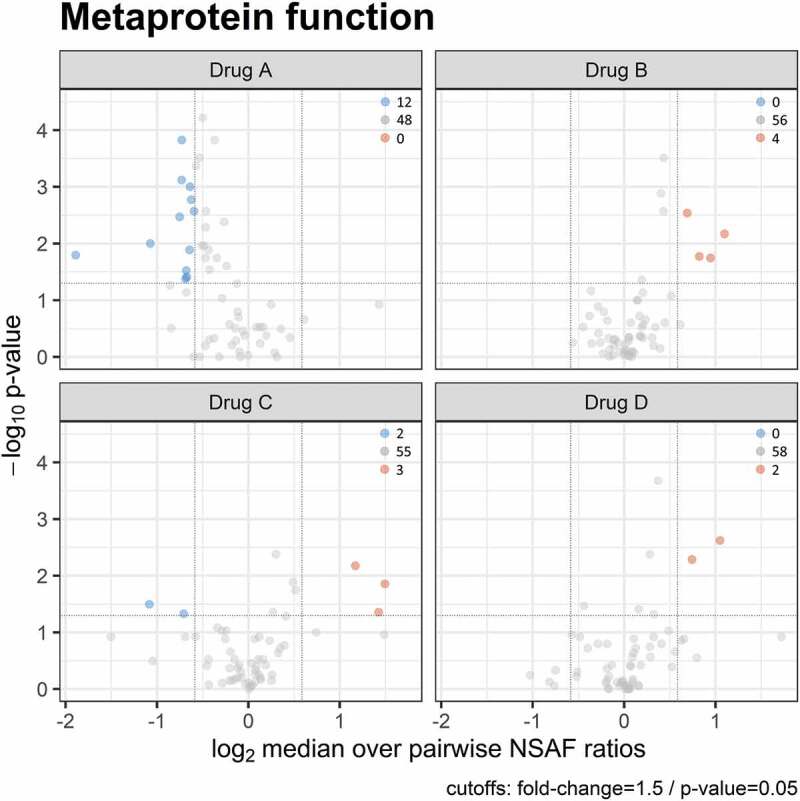
Volcano plot showing significant changes of a paired two-sided Wilcoxon signed rank test (confidence interval = 0.95) for the bacterial metaprotein functions for all four treatments based on the subrole level of the TIGRFAM classification. Blue indicates a significant reduction in relative metaprotein abundance after treatment, red indicates a significant increase, and gray indicates no significant changes in relative metaprotein abundance after treatment.

Metaproteomics enables the measurement and analysis of bacterial proteins, also allowing conclusions regarding interactions between microbes, functional properties of the community as well as to responses to changing environmental conditions [46]. Using a paired two-sided Wilcoxon signed rank test (confidence interval = 0.95; cut-offs: fold-change = 1.5; p-value = 0.05), we evaluated whether significant changes of abundance for metaprotein functions were detectable. Therefore, we analyzed all bacterial metaproteins with respect to their functional classification, which was based on the TIGRFAM system including three levels of classification, which differ in their granularity. One thousand three hundred and sixteen TIGRFAMs could be assigned to the bacterial metaproteins, which were distributed among 60 biological processes (Supplemental Table 3). At the lowest level of the TIGRFAM classification, no significant changes were observed. However, significant changes occurred in 19 biological processes, the second level of the TIGRFAM classification (Figure 6 and Table 2). Supplemental Figure 3 summarizes the treatment-related changes for those biological processes.

**Table 2.** Summary of significant changed metaprotein functions under treatment of Drug A (Listerine®, positive control), Drug B (0.083% H202 accordingly a 1:2 H202/SCN- relation), Drug C (0.04% H202 according to a 1:2 H202/SCN- relation) and Drug D (placebo) based on the subrole level of the TIGRFAM classification.

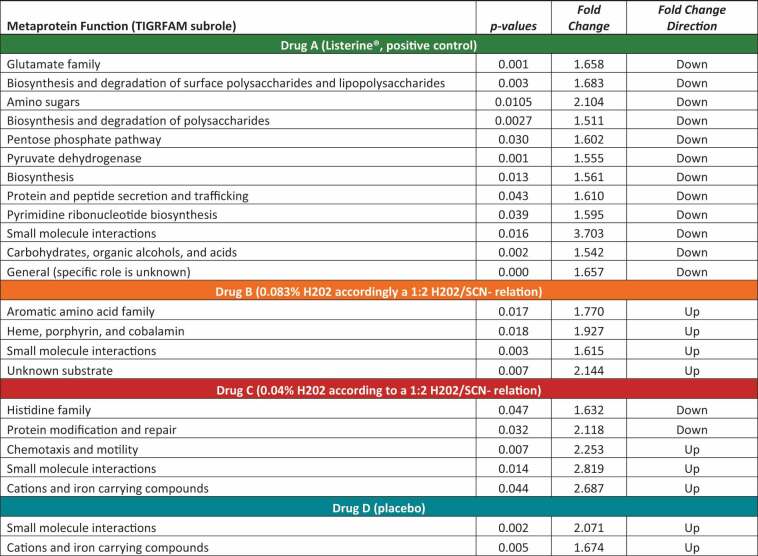

The most significant changes were observed for Drug A (Listerine®, positive control) under whose treatment metaproteins involved in 12 biological processes (Table 2) showed a reduced metaprotein abundance. Metaproteins involved in small-molecule interactions (PAS domain S-box protein [47]) mainly of the category ‘amino sugars’ [48,49] like glucosamine-6-phosphate deaminase, phospho-glucosamine mutase or N-acetylglucosamine-6-phosphate deacetylase showed the most significant differences between control and treatment.

For Drug B (0.083% H_2_0_2_; 1:2 H_2_0_2_/SCN^−^ relation), a significant increase in abundance was observed for metaproteins of the aromatic amino acid family, small-molecule interactions, iron metabolism, and for metabolism of unknown substrates (Table 2). Drug C (0.04% H_2_0_2_; 1:4 H_2_0_2_/SCN^−^ relation) and Drug D (placebo) both showed an increase in abundance for proteins of the small-molecule interactions (PAS domain S-box protein [47]) and cations and iron carrying compounds like bacterioferritin, ubiquinone oxidoreductase or TonB-dependent siderophore receptor [50,51] (Table 2), with an additional increase in chemotaxis and motility, e.g, flagellar M-ring protein (FliF) or flagellar motor switch protein (FliM) [52] for Drug C (0.04% H_2_0_2_; 1:4 H_2_0_2_/SCN^−^ relation). Furthermore, for Drug C, we observed a reduced abundance for proteins of the histidine family (histidinol dehydrogenase, phosphoribosyl-ATP diphosphatase) and protein modification and repair (methionine aminopeptidase [53], L-isoaspartate O-methyltransferase [54]).

Overall, it can be concluded that for Drug A (Listerine®, positive control) most significant changes were observed for biological processes accompanied by a reduction in abundance. For Drugs B (0,083% H_2_0_2_; 1:2 H_2_0_2_/SCN^−^ relation), C (0,04% H_2_0_2_; 1:4 H_2_0_2_/SCN^−^ relation), and D (placebo), the number of significant changes was lower, but always showed an increase in abundance with two exceptions for Drug C. The small-molecule interactions were common to all treatments and were present in reduced abundance for Drug A (positive control) and with an increased abundance for Drugs B, C, and D (placebo).

## Discussion

In this pilot study, metaproteomic techniques are used for the first time to evaluate the influence of a conventional antiseptic in comparison to an antimicrobial human defense system on supragingival plaque formation based on a standardized and widely accepted study model in dentistry [20]. For our study, we used Drug A (Listerine®, positive control) and Drug D (placebo) as positive and negative controls, to directly attribute the changes of the plaque-microbiome to the components of the LPO system, which was included in Drug B (0.083% H_2_0_2_; 1:2 H_2_0_2_/SCN^−^ relation) and Drug C (0.04% H_2_0_2_; 1:4 H_2_0_2_/SCN^−^ relation) with different concentrations of hydrogen peroxide and in H_2_0_2_/SCN^−^ relation.

We benchmarked our results with the number of protein identifications and identified genera with the current literature. Compared to previous studies [55–58], we achieved higher protein identifications with 1,916 (± 465) bacterial metaproteins and 442 (± 171) human proteins per sample. One aspect to consider is that, with 16 subjects and 128 measured samples, we included more subjects and analyzed substantially more samples than comparable metaproteomic studies [55–58]. Further more, there are combined effects of a different sample preparation protocol as well as up-to-date mass spectrometers and data analysis strategies [59–61].

Bacterial metaproteins accounted for the largest proportion with on average three-quarters of the sample in comparison to human proteins. Since we scraped a biofilm from the supragingival area, the high level of bacterial proteins was to be expected, as a biofilm mainly consists of bacteria, extracellular polymeric substance (EPS) as well as other organic and inorganic components like Ca, Mg, SO_4_, lipids or nucleic acids [4,62–64].

Upon exposure to Drug A (Listerine®, positive control) the relative abundance of bacterial metaproteins in total decreased, whereas the relative abundance of human proteins increased accordingly. Probably this is due to the inhibitory effect of Drug A (Listerine®, positive control) on plaque formation in general [65–67]. Drug A (Listerine®, positive control) was a commercially available antiseptic mouth rinse, whose bactericidal effect is based on essential oils and ethanol [68]. In our clinical part of the study, a reduced biofilm was also demonstrated by the observed median QHI value of 0.88 after treatment [19]. This was reflected in fewer identified bacterial metaproteins and their relative abundance, which was paralleled in changes in taxonomic and functional assignment. We identified the most significant reductions in small-molecule interactions, such as the PAS domain S-box protein [47], which plays a role in various signaling processes, such as histidine kinases or chemotaxis. Amino sugars, also with one of the highest reductions, are an important component of the peptidoglycan of the cell wall of bacteria and at the same time a source of energy, nitrogen, and carbon via their degradation [48,49]. In summary, a significant reduction in several metabolic processes mostly affecting key metabolic pathways for growth and proliferation of bacterial cells occurred, which suggests a reduced growth of the bacterial populations after Listerine® treatment.

In contrast, we observed a slight increase in metaprotein abundance and identification with the other three treatments. The results indicate that there may be increased bacterial activity in the biofilm. The increased abundances of flagellar proteins (FliF, FliM) indicating the movement of, for example, still present planktonic initial colonizers moving chemotactically down the nutrient gradient (PAS domain S-box protein [47]) [52]. Another example is the TonB-dependent siderophore receptor relevant for iron supply to bacteria [51], which transports iron from the environment into the cell for deoxyribonucleotide synthesis or oxidative phosphorylation [50]. Another indication is the increased metaprotein abundances of proteins involved in the repair or degradation of damaged proteins (methionine aminopeptidase [53], L-isoaspartate O-methyltransferase [54]). Additionally, based on the median QHI for Drug B (QHI 1.6), Drug C (QHI 1.8), and Drug D (QHI 2.6) a less inhibitory effect on plaque formation could be determined [19].

Regarding the taxonomic diversity, phyla such as *Actinobacteria, Firmicutes* or *Fusobacteria* dominated the assignment of metaproteins and confirmed the results of previous studies [69–74]. The same applies to the genus level, where e.g. *Actinomyces* and *Streptococcus* are among the most represented genera [56,73,75,76]. However, the species level offers the greatest information content for dental practitioners, especially regarding the colonization of tooth surfaces by initial and secondary colonizers [77,78].

The metaprotein abundances and their assigned species showed only small changes after treatment with Drug D (placebo). There were a few significant changes, e.g. for increased metaprotein abundances of the secondary colonizers *Capnocytophaga spp* [77,79,80]. Functionally, the abundance of metaproteins in the categories of small-molecule interactions as well as the cations and iron carrying compounds increased. Drug D was designed as a placebo, and therefore we did not expect many significant changes in the metaproteome. We assume a slight influence by the sugar alcohols mannitol, sorbitol and xylitol contained in Drug D (placebo). Previous studies have already provided initial evidence that sugar alcohols can also have an influence on bacteria and their growth [81–86].

Drugs B and C contained all components of the LPO system with an equal level of LPO concentration whereas Drug B (0.083% H_2_0_2_; 1:2 H_2_0_2_/SCN^−^ relation) contained the hydrogen peroxide donor CPO at a higher concentration than Drug C (0.04% H_2_0_2_; 1:4 H_2_0_2_/SCN^−^ relation).

Drug C had a minor effect on the plaque microbiome and the data generated are comparable to the results of Drug D (placebo). As an example, we also found higher metaprotein abundances for similar species, such as the secondary colonizer *Capnocytophaga spp., Neisseria flava*, or *Leptotrichia sp* [77–80]. Therefore, we suggest that the low concentration of CPO is not sufficient to make a decisive contribution to the growth of the plaque biofilm that goes beyond the effect of the placebo.

A decisive influence on the plaque metaproteome could be observed for Drug B (0.083% H_2_0_2_; 1:2 H_2_0_2_/SCN^−^ relation) especially for metaproteins of beneficial species as well as first and second colonizers. As one example we detected an increased metaprotein abundance for *Lachnospiraceae ssp*., and *Abiotrophia defectiva*, which are associated with dental health in caries-free children [75,87], whereas we could not find references in the literature for each identified species of Lachnospiraceae (see Table 1). Furthermore, metaprotein abundances of *Streptococcus gordonii* were only found significantly increased after treatment of our healthy subjects with Drug B (0.083% H_2_0_2_; 1:2 H_2_0_2_/SCN^−^ relation). It is one of the first colonizers of the oral cavity [88,89] and thus involved in the initial attachment to tooth surfaces and co-aggregates with a variety of bacteria. This bacterium has been further described to compete effectively with *Streptococcus mutans* due to the availability of oxygen and the production of hydrogen peroxide [90,91]. Additionally, the abundance of metaproteins of the secondary colonizers *Capnocytophaga spp*., which are described as commensals and associated with disease in the literature, was also elevated after treatment with Drug B (0.083% H_2_0_2_; 1:2 H_2_0_2_/SCN^−^ relation) [92–94]. The bridging species *Fusobacterium nucleatum subsp*., reported to coaggregate with all early and late colonizer, or even the late colonizers *Prevotella intermedia* and *Prevotella nigrescens* showed also higher metaprotein abundances after treatment with Drug B (0.083% H_2_0_2_; 1:2 H_2_0_2_/SCN^−^ relation) [77–80] both associated with periodontitis [95,96]. In summary, we identified positive changes regarding metaprotein abundances of health-associated bacteria for caries, but negative changes occurred in periodontitis-associated bacteria.

During the complex process of the development of dental caries, an increase in acidogenic bacteria like *Streptococcus mutans* is associated with an ecologic shift in the oral biofilm [88,90]. The treatment period extended over a duration of 4 days to allow a regrowth of the plaque biofilm but was too short to produce a shift of the biofilm towards a diseased status [97–100]. Therefore, no metaproteins from pathogenic species were expected. A more detailed analysis showed that we identified only 11 metaproteins for *S. mutans*, with only one metaprotein being statistically relevant because it was found in more than 50% of all samples. For this single identifier only, we found increased metaprotein abundances for *Streptococcus mutans*, not only after the treatment for Drug B (0.083% H_2_0_2_; 1:2 H_2_0_2_/SCN^−^ relation) but also for Drug C (0.04% H_2_0_2_; 1:4 H_2_0_2_/SCN^−^ relation) and Drug A (Listerine®, positive control). The metaprotein (identifier: smut_c_1_284) is a dehydrogenase in lipid metabolism that has not yet been further characterized. In comparison, we identified considerably more metaproteins for other species, such as for *S. gordonii* with 60 metaproteins or *F. nucleatum* with 235 metaproteins. In addition, other omics studies also identified pathogenic species in healthy subjects [101,102] and is consistent with the extended ecological plaque hypothesis [103,104]. Another point to consider is that the metaprotein abundances of the 10 days recovery phase including 3 days of absence of any oral hygiene procedure on day 3 (baseline oral biofilm) are already at a relatively high level. This baseline oral biofilm was just influenced by a test substance for the following 4 days without other oral hygiene procedures.

A unique challenge was to reconcile the results of the clinical part of the study with the results of the metaproteomic approach. A direct comparison of the observed QHI values of the clinical study [19] and the relative protein amounts (NSAF values) calculated in this metaproteomic study might be misleading because for all samples the same protein amounts were used for MS-based profiling even if treatments had different effects on total biofilm amount (QHI values). Nevertheless, we consider the combination of classical microbiological methods with metaproteomic data in addition with clinical parameters as a valuable approach. By integrating a multi-OMICs approach in the future, we expect to gain even deeper insights into the pathophysiology of dental disease.

## Conclusion

Although the study of molecular mechanisms in complex biofilms using metaproteomic approaches is still in its infancy, we were able to elucidate the impact of four treatments on the plaque metaproteome and associate it with clinical parameters. It could be shown that the metaproteomic analyses not only contribute to the elucidation of the taxonomic composition but also gather functional information for the plaque biofilm during treatment.

According to the data of this metaproteomic analysis, we were able to show that the treatment based on the components of the LPO system induces a change in the plaque metaproteome that differs from that of a placebo and Listerine®. While the reduction of the Quigley-Hein index shown in the clinical study [19] for the antiseptics can be attributed to a reduction in the overall microbiome, our results suggest that the plaque reduction of the LPO-lozenges based more on an increase in bacterial diversity.

## Supplementary Material

Supplemental Material

## Abbreviations

CPO – carbamide peroxide; EPS – extracellular polymeric substance; DTT – dithiothreitol; FDR – false discovery rate; EDTA – ethylenediaminetetraacetic acid; HOMD – Human Oral Microbiome Database; HPLC – high performance liquid chromatography; H_2_O_2_ – Hydrogen peroxide; IAA – iodo acetamide; LCA – Lowest-Common-Ancestor Algorithm; LPO – Lactoperoxidase; MS – mass spectrometry; MS/MS – tandem mass spectrometry; nLC – nano liquid chromatography; NSAF – normalized spectral abundance factor; OSCN^−^ – hypothiocyanite; pepFDR – peptide false discovery rate; protFDR – protein false discovery rate; SCN^−^ – thiocyanate; TCA – trichloroacetic acid; TE – Tris/EDTA; TIGRFAM - database of protein families
