# Supplementary material for: Impact of different oral treatments on the composition of the supragingival plaque microbiome
Source: J Oral Microbiol. 2022 Oct 31;14(1):2138251. doi: 10.1080/20002297.2022.2138251 (PMC9629129; doi:10.1080/20002297.2022.2138251)
Supplement: Supplemental Material [file ZJOM_A_2138251_SM0439.zip › Supplementary/Supplementary figure legend.docx]

**Supplemental Figure 1**

Boxplot showing the changes regarding the identified bacterial metaproteins in percent. The time points after treatment (D7) refer to their corresponding control time points (D3), which are the baseline.

**Supplemental Figure 2**

Violin plot showing significant changes of a paired two-sided Wilcoxon signed rank test (confidence interval = 0.95) for the metaprotein abundances assigned on the genus level for all four treatments.

**Supplemental Figure 3**

Violin plot showing significant changes of a paired two-sided Wilcoxon signed rank test (confidence interval = 0.95) for the bacterial metaprotein functions for all four treatments based on the subrole level of the TIGRFAM classification.
