# Supplementary material for: Impact of different oral treatments on the composition of the supragingival plaque microbiome
Source: J Oral Microbiol. 2022 Oct 31;14(1):2138251. doi: 10.1080/20002297.2022.2138251 (PMC9629129; doi:10.1080/20002297.2022.2138251)
Supplement: Supplemental Material [file ZJOM_A_2138251_SM0439.zip › Supplementary/Supplementary table.docx]

**Supplemental Table 1: Summary of significant changed metaprotein abundances and their taxonomic assignment on the species level under Drug A (Listerine®, positive control).** **For each species, their association with healthy and/or diseased oral conditions is given and is color-coded for visual support.**

**Color legend: green - commensal or health associated; yellow – commensal and disease associated; red: disease associated; grey: no information available if the species is commensal, health or disease associated**

| ***Species*** | ***p-values*** | ***Fold Change*** | ***Fold Change Direction*** | ***Health/Disease Association*** |
| --- | --- | --- | --- | --- |
| *Porphyromonas catoniae* | 0.016 | 3.529 | Down | Health ^(128)^ |
| *Porphyromonas sp. oral taxon 279* | 0.000 | 5.096 | Down | Disease associated genus ^(129)^ |
| *Porphyromonas sp. OT 278 W7784* | 0.005 | 4.065 | Down | Disease associated genus ^(129)^ |
| *Tannerella sp. oral taxon BU063 isolate Cell 5* | 0.008 | 2.756 | Down | Unknown |
| *Prevotella sp. oral taxon 317* | 0.043 | 3.609 | Down | Unknown |
| *Prevotella sp. oral taxon 473 F0040* | 0.014 | 9.274 | Down | Unknown |
| *Capnocytophaga sp. oral taxon 329 F0087* | 0.006 | 2.476 | Down | Commensal and Disease ^(109)^ |
| *Capnocytophaga sputigena* | 0.016 | 1.730 | Down | Commensal and Disease ^(109,123)^ |
| *Gemella morbillorum* | 0.038 | 1.674 | Down | Commensal and Disease ^(130,131)^ |
| *Streptococcus peroris* | 0.000 | 2.037 | Down | Commensal and Disease ^(114,115)^ |
| *Catonella morbi* | 0.002 | 1.971 | Down | Disease ^(132)^ |
| *Lachnospiraceae bacterium Oral taxon 107 F0167* | 0.039 | 2.320 | Down | Unknown |
| *Lachnospiraceae bacterium sp. oral taxon 082 F0431* | 0.025 | 1.777 | Down | Unknown |
| *Peptostreptococcaceae saburreum ATCC 49989* | 0.011 | 2.252 | Down | Unknown |
| *Leptotrichia buccalis* | 0.002 | 2.017 | Down | Commensal and Disease ^(120,121)^ |
| *Leptotrichia goodfellowii* | 0.012 | 3.979 | Down | Commensal and Disease ^(120,121,133)^ |
| *Leptotrichia hofstadii* | 0.008 | 2.732 | Down | Commensal and Disease ^(120,121)^ |
| *Leptotrichia shahii* | 0.004 | 2.027 | Down | Commensal and Disease ^(120,121)^ |
| *Leptotrichia sp. oral taxon 215* | 0.002 | 2.856 | Down | Commensal and Disease ^(120,121)^ |
| *Leptotrichia sp. oral taxon 225* | 0.000 | 2.577 | Down | Commensal and Disease ^(120,121)^ |
| *Leptotrichia wadei* | 0.001 | 2.023 | Down | Commensal and Disease ^(120,121)^ |
| *Kingella oralis* | 0.010 | 2.763 | Down | Unknown |
| *Neisseria flava* | 0.025 | 4.589 | Down | Commensal and Health ^(124–126)^ |
| *Neisseria sp. oral taxon 014 F0314* | 0.003 | 1.656 | Down | Unknown |
| *Neisseria sp. oral taxon 020* | 0.023 | 8.258 | Down | Unknown |
| *Cardiobacterium hominis* | 0.001 | 2.037 | Down | Disease ^(134)^ |
| *Aggregatibacter aphrophilus* | 0.005 | 18.216 | Down | Commensal and Disease ^(135)^ |
| *Haemophilus influenzae* | 0.000 | 2.610 | Down | Commensal and Disease ^(135)^ |
| *Rothia aeria* | 0.005 | 1.934 | Up | Disease ^(104)^ |
| *Rothia dentocariosa* | 0.004 | 5.128 | Up | Commensal and Disease ^(105–107)^ |
| *Streptococcus mutans* | 0.000 | 3.120 | Up | Commensal and Disease ^(114,115)^ |
| *Streptococcus pneumoniae* | 0.034 | 2.534 | Up | Commensal and Disease ^(114,115)^ |
| *Streptococcus sp. oral taxon 058* | 0.039 | 1.600 | Up | Unknown |
| *Peptoniphilus sp. oral taxon 375* | 0.001 | 2.636 | Up | Disease associated genus ^(136)^ |
| *Pyramidobacter piscolens* | 0.045 | 3.487 | Up | Unknown ^(137)^ |

**Supplemental Table 2: Summary of significant changed metaprotein abundances and their taxonomic assignment on the species level under Drug D (placebo). For each species, their association with healthy and/or diseased oral conditions is given and is color-coded for visual support.**

**Color legend: green - commensal or health associated; yellow – commensal and disease associated; red: disease associated; grey: no information available if the species is commensal, health or disease associated**

| ***Species*** | ***p-values*** | ***Fold Change*** | ***Fold Change Direction*** | ***Health/Disease Association*** |
| --- | --- | --- | --- | --- |
| *Actinomyces sp. oral taxon 448* | 0.029 | 1.626 | Down | Unknown |
| *Rothia aeria* | 0.021 | 1.838 | Down | Disease ^(104)^ |
| *Rothia dentocariosa* | 0.029 | 2.586 | Down | Commensal and Disease ^(105–107)^ |
| *Corynebacterium durum* | 0.021 | 2.896 | Down | Commensal ^(108)^ |
| *Actinomyces odontolyticus* | 0.018 | 3.940 | Up | Disease ^(138,139)^ |
| *Actinomyces sp. oral taxon 180* | 0.008 | 1.992 | Up | Unknown |
| *Prevotella oulorum* | 0.029 | 4.328 | Up | Unknown |
| *Capnocytophaga gingivalis* | 0.029 | 1.604 | Up | Commensal and Disease ^(109,140)^ |
| *Capnocytophaga sp. oral taxon 326* | 0.004 | 1.945 | Up | Commensal and Disease ^(109)^ |
| *Capnocytophaga sp. oral taxon 329 F0087* | 0.003 | 1.610 | Up | Commensal and Disease ^(109)^ |
| *Capnocytophaga sp. oral taxon 332* | 0.001 | 2.974 | Up | Commensal and Disease ^(109)^ |
| *Capnocytophaga sputigena* | 0.001 | 2.662 | Up | Commensal and Disease ^(109,123)^ |
| *Clostridiales [F-1] [G-1] sp. oral taxon 876 F0540* | 0.021 | 2.487 | Up | Unknown |
| *Leptotrichia sp. oral taxon 215* | 0.002 | 1.811 | Up | Commensal and Disease ^(120,121)^ |
| *Neisseria flava* | 0.004 | 1.983 | Up | Commensal and Health ^(124–126)^ |

Supplemental Table 3: Summary of all identified changed metaprotein functions under treatment of Drug A (Listerine®, positive control), Drug B (0.083% H202 accordingly a 1:2 H202/SCN- relation), Drug C (0.04% H202 according to a 1:2 H202/SCN- relation), Drug D (placebo) based on the subrole level of the TIGRFAM classification.

|  | **Drug A**  **(Listerine®,**  **positive control)** | | **Drug B**  **(0.083% H202 accordingly a 1:2 H202/SCN- relation)** | | **Drug C**  **(0.04% H202 according to a 1:2 H202/SCN- relation)** | | **Drug D**  **(placebo)** | |
| --- | --- | --- | --- | --- | --- | --- | --- | --- |
| **Metaprotein Function (TIGRFAM subrole)** | ***Fold Change*** | ***Fold Change Direction*** | ***Fold Change*** | ***Fold Change Direction*** | ***Fold Change*** | ***Fold Change Direction*** | ***Fold Change*** | ***Fold Change Direction*** |
| Aromatic amino acid family | 1.373 | up | 1.770 | up | 2.065 | down | 1.739 | up |
| Aspartate family | 1.485 | down | 1.080 | down | 1.190 | down | 1.138 | down |
| Glutamate family | 1.658 | down | 1.028 | up | 1.021 | down | 1.051 | up |
| Histidine family | 1.223 | up | 1.300 | down | 1.632 | down | 1.013 | up |
| Pyruvate family | 1.383 | down | 1.032 | up | 1.072 | down | 1.050 | up |
| Serine family | 1.104 | down | 1.071 | down | 1.033 | up | 1.488 | down |
| Heme, porphyrin, and cobalamin | 1.099 | down | 1.927 | up | 1.370 | down | 1.121 | down |
| Menaquinone and ubiquinone | 1.242 | up | 1.175 | up | 1.353 | down | 3.301 | up |
| Pyridine nucleotides | 1.794 | down | 1.173 | down | 1.615 | down | 1.121 | down |
| Riboflavin, FMN, and FAD | 1.814 | down | 1.278 | down | 2.797 | up | 2.038 | down |
| Biosynthesis and degradation of murein sacculus and peptidoglycan | 1.041 | down | 1.316 | up | 1.083 | down | 1.071 | up |
| Biosynthesis and degradation of surface polysaccharides and lipopolysaccharides | 1.683 | down | 1.156 | up | 1.273 | up | 1.217 | up |
| Surface structures | 1.134 | down | 1.040 | up | 1.192 | up | 1.399 | down |
| Adaptations to atypical conditions | 1.190 | up | 1.473 | down | 1.055 | up | 1.697 | down |
| Cell division | 1.119 | up | 1.216 | down | 1.145 | down | 1.577 | up |
| Chemotaxis and motility | 1.386 | down | 1.430 | up | 2.253 | up | 1.255 | up |
| Detoxification | 1.423 | down | 1.038 | up | 1.096 | up | 1.054 | down |
| DNA transformation | 1.247 | down | 1.214 | down | 1.332 | up | 1.683 | down |
| Pathogenesis | 1.447 | down | 1.269 | up | 1.352 | down | 1.134 | up |
| Amino sugars | 2.104 | down | 1.091 | down | 1.254 | up | 1.040 | up |
| Nitrogen metabolism | 1.077 | down | 1.196 | down | 1.431 | up | 1.034 | down |
| Phosphorus compounds | 1.602 | down | 1.285 | down | 1.053 | up | 1.356 | down |
| DNA replication, recombination, and repair | 1.384 | down | 1.047 | down | 1.110 | down | 1.067 | up |
| Aerobic | 1.281 | up | 1.120 | down | 1.223 | down | 1.318 | down |
| Amino acids and amines | 1.396 | down | 1.350 | up | 1.085 | up | 1.119 | up |
| Anaerobic | 1.001 | down | 1.113 | down | 1.054 | up | 1.127 | down |
| Biosynthesis and degradation of polysaccharides | 1.511 | down | 1.082 | down | 1.152 | down | 1.111 | up |
| Electron transport | 1.091 | down | 1.323 | up | 1.118 | up | 1.295 | up |
| Fermentation | 1.291 | down | 1.101 | up | 1.022 | up | 1.042 | up |
| Glycolysis/gluconeogenesis | 1.202 | down | 1.052 | up | 1.064 | up | 1.008 | up |
| Pentose phosphate pathway | 1.602 | down | 1.361 | up | 1.080 | up | 1.545 | up |
| Pyruvate dehydrogenase | 1.555 | down | 1.006 | up | 1.232 | up | 1.252 | up |
| Sugars | 1.270 | down | 1.135 | down | 1.001 | up | 1.220 | down |
| TCA cycle | 1.380 | down | 1.080 | up | 1.404 | up | 1.077 | up |
| Biosynthesis | 1.561 | down | 1.348 | up | 1.205 | up | 1.216 | up |
| Conserved | 1.062 | down | 1.059 | up | 1.099 | up | 1.009 | up |
| Degradation of proteins, peptides, and glycopeptides | 1.094 | up | 1.252 | up | 1.132 | down | 1.051 | up |
| Protein and peptide secretion and trafficking | 1.610 | down | 1.060 | up | 1.674 | up | 1.473 | up |
| Protein folding and stabilization | 1.412 | down | 1.036 | up | 1.058 | down | 1.012 | down |
| Protein modification and repair | 1.295 | down | 1.036 | up | 2.118 | down | 1.095 | down |
| Ribosomal proteins: synthesis and modification | 1.351 | down | 1.001 | up | 1.175 | up | 1.032 | up |
| Translation factors | 1.181 | down | 1.143 | up | 1.050 | up | 1.094 | up |
| tRNA aminoacylation | 1.083 | down | 1.155 | up | 1.021 | up | 1.221 | up |
| tRNA and rRNA base modification | 2.696 | up | 1.123 | down | 1.199 | up | 1.758 | down |
| Nucleotide and nucleoside interconversions | 1.027 | down | 1.154 | up | 1.018 | up | 1.076 | up |
| Purine ribonucleotide biosynthesis | 1.447 | down | 1.110 | up | 1.084 | up | 1.401 | up |
| Pyrimidine ribonucleotide biosynthesis | 1.595 | down | 1.531 | up | 1.108 | down | 1.327 | up |
| Salvage of nucleosides and nucleotides | 1.343 | down | 1.237 | up | 1.265 | down | 1.022 | down |
| DNA interactions | 1.092 | up | 1.125 | up | 1.083 | down | 1.433 | down |
| Small molecule interactions | 3.703 | down | 1.615 | up | 2.819 | up | 2.070 | up |
| PTS | 1.153 | down | 1.144 | up | 1.141 | up | 1.066 | up |
| Degradation of RNA | 1.346 | down | 1.364 | down | 1.316 | up | 1.120 | up |
| Transcription factors | 1.218 | down | 1.133 | up | 1.156 | down | 1.010 | down |
| Amino acids, peptides, and amines | 1.069 | down | 1.008 | up | 2.838 | down | 1.426 | down |
| Anions | 1.515 | down | 1.004 | up | 1.006 | up | 1.034 | up |
| Carbohydrates, organic alcohols, and acids | 1.542 | down | 1.165 | down | 1.185 | down | 1.137 | down |
| Cations and iron carrying compounds | 1.146 | up | 1.037 | down | 2.687 | up | 1.674 | up |
| Unknown substrate | 1.065 | up | 2.144 | up | 1.219 | down | 1.438 | up |
| Enzymes of unknown specificity | 1.525 | up | 1.065 | up | 1.493 | down | 1.146 | down |
| General | 1.657 | down | 1.127 | up | 1.052 | down | 1.141 | down |

**Supplemental Table 4: Listing and references of the used R-packages (39).**

| ***Name of R package*** | ***Short description of R package*** | ***Link*** |
| --- | --- | --- |
| ape | Analyses of phylogenetics and evolution | [https://CRAN.R-project.org/package=ape](https://cran.r-project.org/package=ape) |
| data.table | Extension of 'data-frame' | [https://CRAN.R-project.org/package=data.table](https://cran.r-project.org/package=data.table) |
| ggplot2 | Create elegant data visualizations using grammar of graphics | [https://CRAN.R-project.org/package=ggplot2](https://cran.r-project.org/package=ggplot2) |
| ggpubr | 'ggplot2' based publication ready plots | [https://CRAN.R-project.org/package=ggpubr](https://cran.r-project.org/package=ggpubr) |
| ggrepel | Automatically position non-overlapping text labels with 'ggplot2' | [https://CRAN.R-project.org/package=ggrepel](https://cran.r-project.org/package=ggrepel) |
| ggsignif | Significance brackets for 'ggplot2' | [https://CRAN.R-project.org/package=ggsignif](https://cran.r-project.org/package=ggsignif) |
| helfRlein | General helper functions that ease our programing life, daily work, and projects | <https://github.com/STATWORX/helfRlein> |
| metacoder ^(48)^ | Tools for parsing, manipulating, and graphing taxonomic abundance data | [https://CRAN.R-project.org/package=metacoder](https://cran.r-project.org/package=metacoder) |
| patchwork | The composer of plots | [https://CRAN.R-project.org/package=patchwork](https://cran.r-project.org/package=patchwork) |
| reshape | Flexibly reshape data | [https://CRAN.R-project.org/package=reshape](https://cran.r-project.org/package=reshape) |
| scales | Scale functions for visualization | [https://CRAN.R-project.org/package=scales](https://cran.r-project.org/package=scales) |
| Seqinr ^(136)^ | Biological sequences retrieval and analysis | [https://CRAN.R-project.org/package=seqinr](https://cran.r-project.org/package=seqinr) |
| stringr | Simple, consistent wrappers for common string operations | [https://CRAN.R-project.org/package=stringr](https://cran.r-project.org/package=stringr) |
| tidyr | Tidy messy data | [https://CRAN.R-project.org/package=tidyr](https://cran.r-project.org/package=tidyr) |
| tidyverse | Easily install and load the 'Tidyverse' | [https://CRAN.R-project.org/package=tidyverse](https://cran.r-project.org/package=tidyverse) |
