# Supplementary figures and images for: Impact of different oral treatments on the composition of the supragingival plaque microbiome
Source: J Oral Microbiol. 2022 Oct 31;14(1):2138251. doi: 10.1080/20002297.2022.2138251 (PMC9629129; doi:10.1080/20002297.2022.2138251)

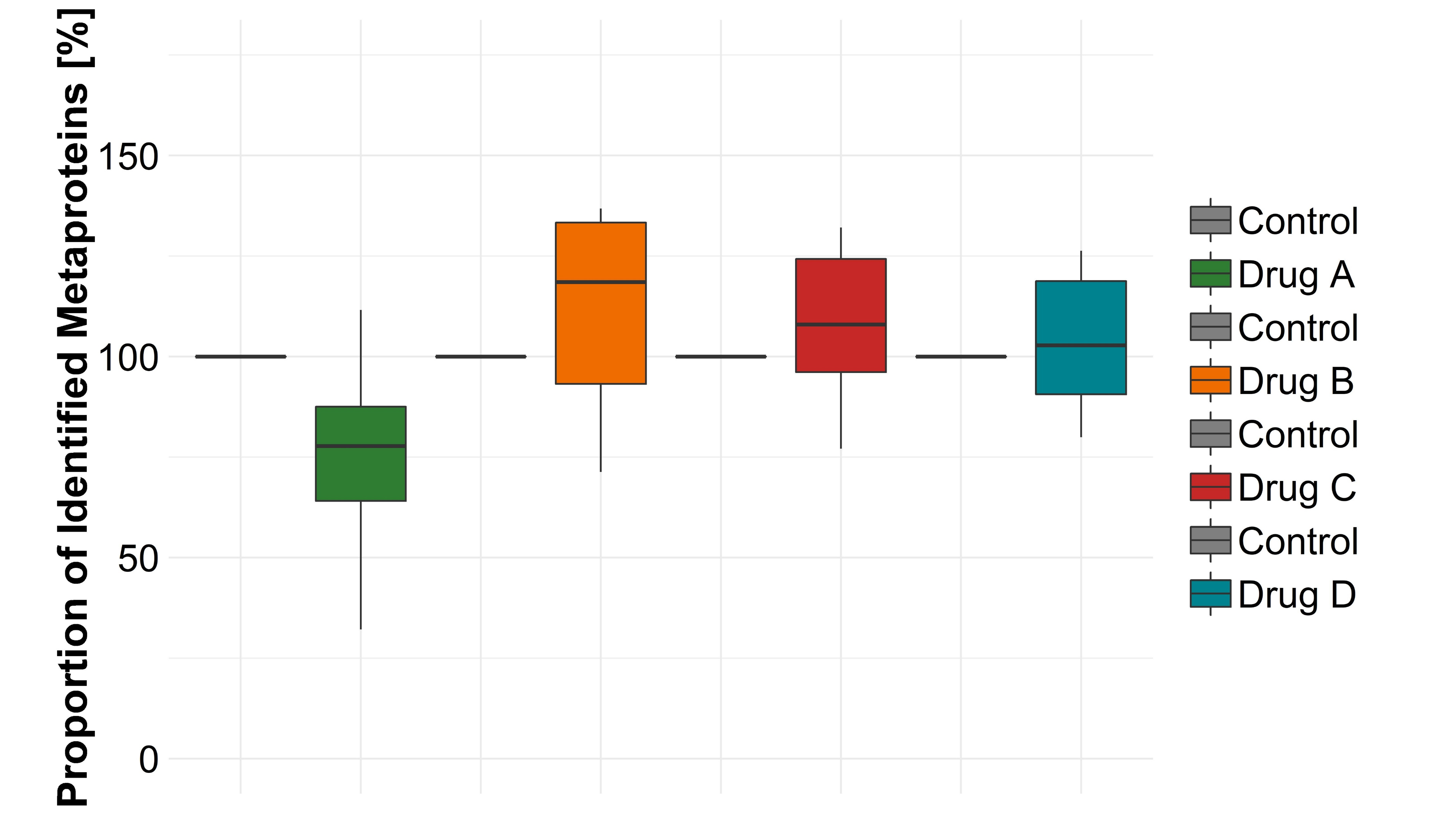

Supplement: Supplemental Material [file ZJOM_A_2138251_SM0439.zip › Supplementary/Supplemental_Figure_1_LPO_study_JoOM.tif]
